# Framework for Classifying Explainable Artificial Intelligence (XAI) Algorithms in Clinical Medicine

**DOI:** 10.2196/50934

**Published:** 2023-09-01

**Authors:** Thomas Gniadek, Jason Kang, Talent Theparee, Jacob Krive

**Affiliations:** 1 Department of Pathology and Laboratory Medicine NorthShore University Health System Evanston, IL United States; 2 Department of Biomedical and Health Information Sciences University of Illinois at Chicago Chicago, IL United States; 3 Department of Health Information Technology NorthShore University Health System Evanston, IL United States; 4 Department of Health Informatics Dr Kiran C Patel School of Osteopathic Medicine Nova Southeastern University Fort Lauderdale, FL United States; 5 Pritzker School of Medicine University of Chicago Chicago, IL United States

**Keywords:** explainable artificial intelligence, XAI, artificial intelligence, AI, AI medicine, pathology informatics, radiology informatics

## Abstract

Artificial intelligence (AI) applied to medicine offers immense promise, in addition to safety and regulatory concerns. Traditional AI produces a core algorithm result, typically without a measure of statistical confidence or an explanation of its biological-theoretical basis. Efforts are underway to develop explainable AI (XAI) algorithms that not only produce a result but also an explanation to support that result. Here we present a framework for classifying XAI algorithms applied to clinical medicine: An algorithm’s clinical scope is defined by whether the core algorithm output leads to observations (eg, tests, imaging, clinical evaluation), interventions (eg, procedures, medications), diagnoses, and prognostication. Explanations are classified by whether they provide empiric statistical information, association with a historical population or populations, or association with an established disease mechanism or mechanisms. XAI implementations can be classified based on whether algorithm training and validation took into account the actions of health care providers in response to the insights and explanations provided or whether training was performed using only the core algorithm output as the end point. Finally, communication modalities used to convey an XAI explanation can be used to classify algorithms and may affect clinical outcomes. This framework can be used when designing, evaluating, and comparing XAI algorithms applied to medicine.

## Introduction

Algorithmic classifiers like artificial neural networks were first implemented many years ago [1]. Recently, unsupervised neural networks have allowed context-agnostic training and deployment. Without the need to embed a priori knowledge of the real-world system being studied, the use of these applications has expanded rapidly, and there has been much excitement about artificial intelligence (AI) algorithms in nearly every industry, including medicine.

Meanwhile, government policy that incentivizes the use of electronic medical record systems expanded the availability of digital health care information [2]. This created an environment where data analysis, predictive analytics, and ultimately AI can readily influence the interpretation of patient data and potentially prevent errors in real time during the course of clinical care [3]. Along these lines, radiologists, and to a lesser extent pathologists, are increasingly using image analysis algorithms as an assistive technology for image interpretation [4-6]. These technologies, rather than feeding into misconceptions about threats and capabilities of AI, could potentially put radiologists and pathologists at the forefront of purposeful AI innovation [7].

Initially, AI may seem like a threat to health care jobs, removing providers from the decision-making process by introducing algorithms that function as a “black box” [8]. With this perceived threat are concerns about patient safety, some stemming from comparisons to non–health care applications of AI. Like any system, AI is not infallible. For example, early versions of self-driving automobile algorithms may have caused accidents [9].

The practice of clinical medicine remains an “art” where decisions of licensed providers are relied upon to ensure patient safety. Unfortunately, in contrast to transparent, rule-based systems, a trained AI model is not transparent to a clinician [10]. Therefore, there are currently efforts to find a middle ground that combines human involvement and AI in a complementary manner [11]. For example, AI might be used to generate insights not always or easily identified by a human, but a human would still determine their significance [12,13]. In this way, AI becomes a tool used by a clinician.

Multiple countries have passed or proposed regulations on the use of algorithms in clinical medicine. Under the US Food, Drug, and Cosmetic Act, an algorithm can be classified as a “nonregulated medical device” if it meets certain criteria; otherwise, it may represent a regulated medical device. One of the key criteria is whether the algorithm is “intended for the purpose of enabling such health care professional to independently review the basis for such recommendations that such software presents so that it is not the intent that such health care professional rely primarily on any of such recommendations to make a clinical diagnosis or treatment decision regarding an individual patient” [14]. It remains to be seen how the FDA enforces this criterion on a case-by-case basis, and regulations may change over time. Similarly, the UK Department of Health and Social Care has issued robust guidance for best practices in digital health care innovation [15]. One of the key elements of this guidance is transparency about algorithm limitations, algorithm type, and evidence of effectiveness. Because of these regulatory frameworks, concerns about medical malpractice issues, and the general awareness that algorithm predictions are not always correct, there is a growing recognition that AI algorithms should allow health care providers to independently review some form of explanation of their core results [16].

Recently, efforts began to build AI algorithms that allow humans to evaluate the significance of their results, with the goal of better integration and communication between the two. Most notably, the US Defense Advanced Research Projects Agency (DARPA) has called for further development of “explainable artificial intelligence” (XAI) [17]. The core algorithmic result or prediction is provided to the user along with an explanation that is intended to convey insight into the confidence of the core prediction, increase a user’s understanding of the real-world process being studied, or both [18].

With its many benefits, XAI also brings added complexity in the form of process-specific outputs and integration with a subject matter expert end user. Not only does this elevate the importance of partnerships between clinicians and AI developers, it also raises the somewhat paradoxical possibility that algorithms with inferior core predictive power may perform better if the explanations provided result in superior outcomes overall. Furthermore, the clinical decision points supported by XAI as well as the manner in which explanations are provided to the user may differ greatly between algorithms and influence their efficacy. Here, we propose a framework for classifying XAI algorithms in clinical medicine in order to simplify this additional complexity and allow for performance evaluation of XAI in clinical practice.

## Clinical Scope

**Figure 1:**
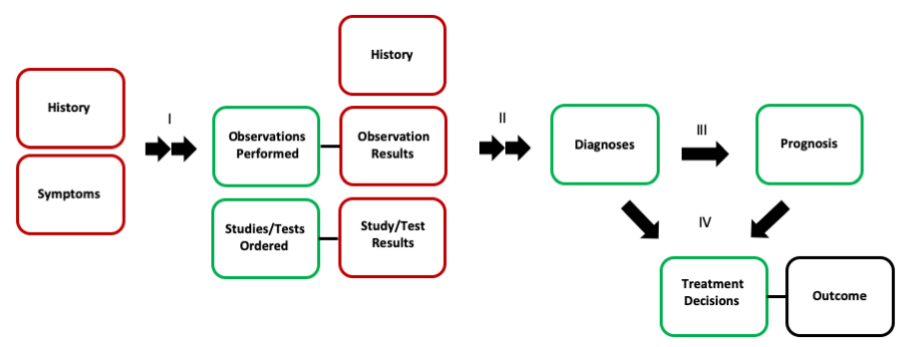
Clinical scope for XAI algorithms. XAI algorithms can be classified based on which steps in the clinical decision-making process they support. A simplified process flow map divides clinical decision-making into information (boxes) and information processing (arrows). Information processing steps (I-IV) can involve both human cognitive processing and computerized algorithms. Disease process evolution introduces biologic time dependency (red boxes), leading to a requirement for repeated information processing over time (double arrows). Some recorded information more directly reflects underlying disease (red boxes), while some is mainly the result of information processing (green boxes). Clinical outcome reflects underlying biology, the performance of the entire process, and the effectiveness of treatments. XAI fundamentally influences the information processing steps (I-IV) in partnership with clinicians. XAI performance can be evaluated at each information processing step or studied in the context of overall outcome. Performance of tests and treatments (black lines) are assumed to be static; however, they can be incorporated as inputs into a decision process. XAI: explainable artificial intelligence.

The ultimate scope of clinical medicine is to prolong and improve the quality of human life. Within this, there are many decisions and actions that can be evaluated independently (eg, ordering a test, prescribing a medication, performing a surgery). XAI algorithms can be classified based on which step(s) in the clinical care pathway they support (see Figure 1). A single algorithm may provide outputs that encompass multiple areas of clinical scope. Defining clinical scope is critical for XAI, because it will determine which individuals on clinical care teams will be best suited to interact with the algorithm and evaluate the explanations provided. Furthermore, the ultimate impact of XAI on clinical outcomes will be limited by the potential impact of the process steps that an algorithm supports.

## Clinical Insight

Explanations provided by XAI algorithms should aim to provide evidence and ultimately insight to the end user. In the case of pathology, generation of insight to assist clinicians can assist with formation of differential diagnoses, quantitative classification of features, risk prediction, and identification of features imperceptible to the human observer [19]. Both the content of the information and its delivery will determine effectiveness. Evidence can be presented in the form of empiric assessments of statistical confidence, such as a *P* value. Alternatively, an algorithm could provide an assessment of the degree of association between the current patient’s data and historical groups of patients or established disease mechanisms (see Table 1).

Clinical providers evaluate empiric assessments of confidence differently than associative power, and the existence of a high degree of uncertainty in any patient-specific medical prediction necessitates a continued role for the “art of medicine” in the form of decision-making by end users. This is due to an incomplete accounting for biological factors that influence disease processes, incomplete documentation of observable factors in the electronic medical record, and the importance of the doctor-patient relationship in clinical care [20]. As a result, associative explanations may be more powerful in certain situations, since an association may support a nonquantifiable opinion held by provider or patient.

**Table 1:** Classifying explainable artificial intelligence explanations by type. The explanations produced by an explainable artificial intelligence algorithm can provide addition information to a clinician in 3 general ways.

XAI^a^ explanation type	XAI explanation output	Primary task for clinician	Benefit to clinician
Empiric	Statistical confidence based on historical sample data	Weigh the degree of confidence provided with risks, benefits, and training data used	Assess the validity of the prediction
Population associative	Association between signs and symptoms of a patient with historical groups of patients	Assess the validity of associating this patient with historical groups of patients	Consider alternative options processed by the algorithm
Mechanism associative	Association with known pathologic mechanism(s)	Assess the validity of the pathologic mechanism(s) and diagnoses proposed	Assess validity of the prediction and consider alternatives using established medical paradigms

^a^XAI: explainable artificial intelligence.

## Training and Validation

The loss of context and end user agnostic efficiency of traditional AI algorithms remains a great challenge to the initial design and implementation of XAI. In fact, the meaning of model validation in medicine differs from the traditional validation process typically undertaken in technology fields in that it refers to validation relative to patient outcomes and evidence-based medicine principles—not just whether outcomes are technically correct, match a reference method, or agree with expectations [21]. Ultimately, only patient outcomes can confirm whether the model is valid and whether AI investment is or was a worthy investment. Therefore, XAI takes special meaning in such evidence-based validation processes, since explainable analytics will help support outcomes or facilitate corrections and adjustments. Likely, the development of context-specific XAI will evolve from traditional AI in phases, each supposing a core algorithm output in addition to some form of explanation: phase I will involve traditional AI training and validation; phase II will involve traditional AI training and XAI validation, taking into account end-user actions; and phase III will involve XAI training and validation, both taking into account end-user actions.

During the final phase of XAI development as described above, the algorithm will train not to maximize the predictive power of the core algorithm output but to maximize the outcome of the combined effects of core output, explanation, and end-user actions. It is during this phase of development that XAI implementations may regain some degree of the context-agnostic advantages of traditional AI, since the behavior of the end-user context expert can be studied by the algorithm during validation.

## Example 1: Anatomic Pathology

**Figure 2:**
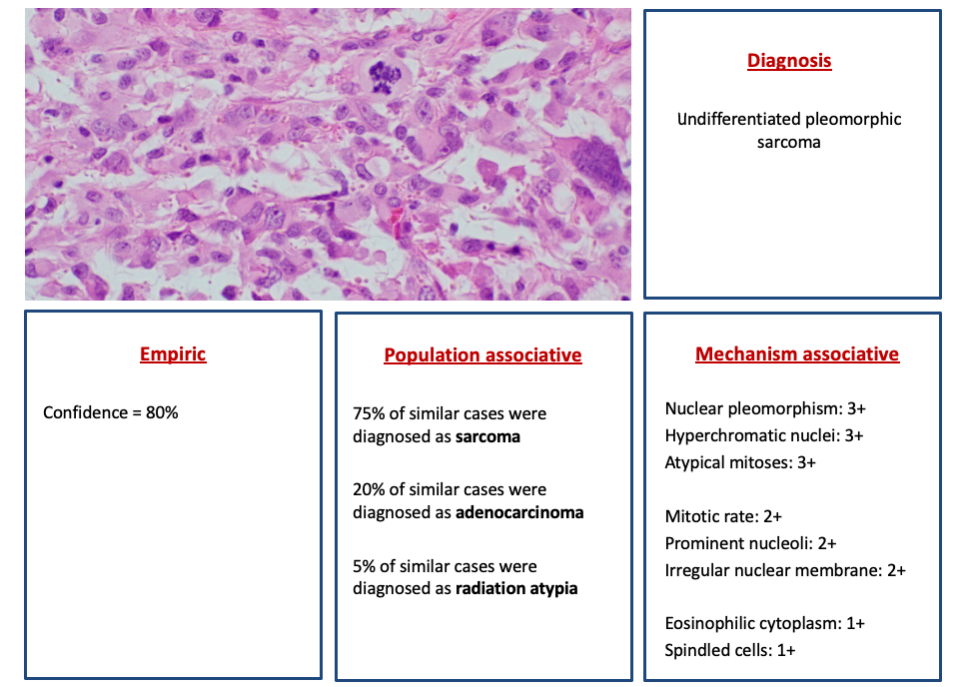
Illustrative example of 3 types of XAI output applied to anatomic pathology. XAI core algorithm output is shown as a diagnosis. Several forms of output explanation are succinctly outlined beneath the image, enabling a physician to make a visual interpretation in conjunction with immediate access to an explanation under multiple categories. “Empiric” information provides overall accuracy expressed as a single number; “population associative” provides a more detailed glimpse into the “black box” result; “diagnosis” relates to other cases an algorithm has access to; “mechanism associative” maps the AI process onto clinically relevant features found in the image (scored based on degree of association, 1 to 3+). XAI: explainable artificial intelligence.

Anatomic pathologists interpret microscopic tissue morphology based on architectural and cytomorphologic criteria shown to correlate with pathologic diagnoses such as cancer. Criteria may include features such as hyperchromatic nuclei, high mitotic rate, and irregular nuclear membrane contours. Unfortunately, none of these features are 100 percent specific for a particular diagnosis like cancer, since nonneoplastic conditions may produce similar cellular features. Additionally, noninvasive premalignant conditions such as carcinoma in situ can contain individual cells that appear morphologically identical to cells within an invasive cancer. Incorporating concepts of XAI into digital anatomic pathology workflows will aid pathologists not only in making the correct diagnosis, but also in considering alternative diagnoses and recognizing potential diagnostic pitfalls (see Figure 2). Potentially, XAI systems can also incorporate ancillary information, such as clinical history, immunohistochemistry staining, and genomic testing, to aid the pathologist.

## Example 2: Diagnostic Management

**Figure 3:**
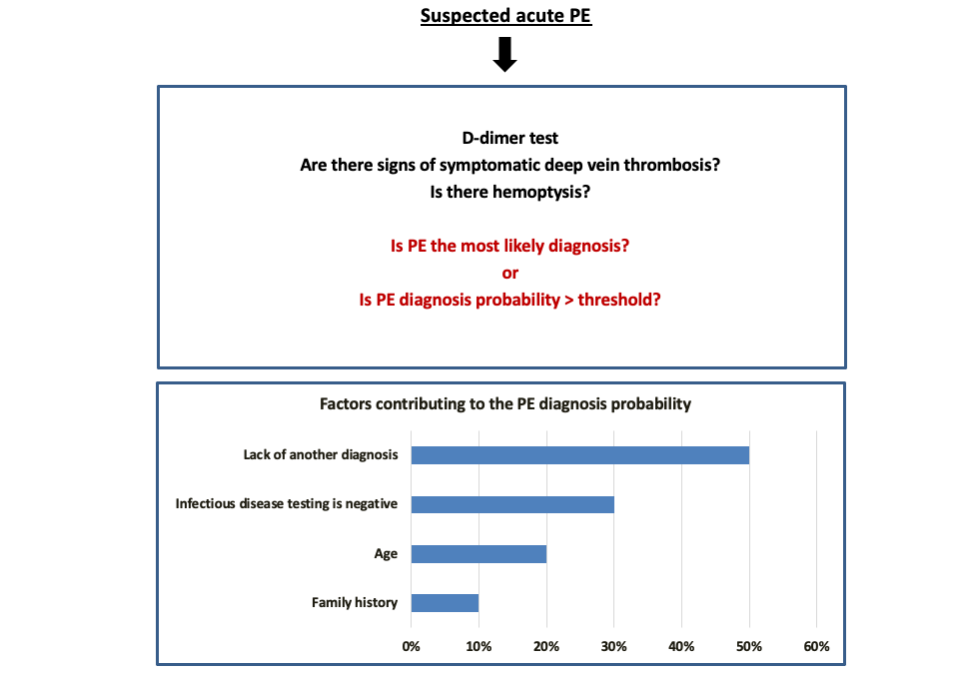
Diagnostic management. Possible modification to the YEARS algorithm for decisions on screening for PE by computed tomography. Rather than relying on clinician assessment of whether PE is the most likely pretest diagnosis, simple scoring algorithms can use an explainable artificial intelligence core algorithm output to assess pretest probability in the context of well-defined historical patient populations. Furthermore, the contribution of factors contributing to the core probability assessment can be displayed. Users can then assess whether each factor is valid, which may influence their assessment of the core algorithm output. For example, factors may be considered invalid if the electronic medical record is recognized as being incomplete or inaccurate. PE: pulmonary embolism.

One of the most difficult tasks for a clinician is to identify which patients should undergo screening tests and which should not [22]. This is particularly difficult when the condition screened for has a high mortality rate if not recognized, but the screening test is expensive and not without risks. Such a situation exists in deciding whether to screen for pulmonary embolism using computed tomography pulmonary angiography [23]. As a result, algorithms have been developed to aid clinical decision-making, but a clinician’s assessment of whether pulmonary embolism is the most likely diagnosis plays a large role in determining a patient’s score and management. Scenarios like this represent an opportunity for XAI to contribute toward more accurate assessments of pretest diagnostic likelihood (see Figure 3).

## Conclusions

The 2 recognized advantages of XAI over traditional AI can be summarized as insight into the statistical significance of a core algorithm output and mechanistic insight into the process being studied. It has been suggested that forcing AI to provide mechanistic understanding could decrease the predictive power of the algorithm itself. This may be true in a situation where algorithm inputs include all data relevant to the real-world process; however, clinical medicine remains an area where digitized information is incomplete relative to the totality of factors influencing human disease. Therefore, humans will likely remain the ultimate “trusted” decision-makers during critical, high-risk decisions in clinical care for the foreseeable future. In this framework, even clinical algorithms that are approved as regulated medical devices will remain ancillary to the human practice of medicine. XAI offers the potential to improve not the predictive power of black box algorithms but rather their usefulness as a tool for clinical providers, offering the opportunity to classify and categorize data [24], as well as ensure meaningful feedback that fits clinical workflows [25]. Information should include identification of tasks, the nature and purpose of the tasks, their outcome, and methods applied to produce the outcome [26].

Medical leaders have discussed the need for a “learning health care system” for many years. The development of XAI offers the potential to build algorithms that learn with clinical care providers. To realize the potential of XAI, we must understand how each type of algorithm might fit into the real-world process of care delivery and the minds of medical decision-makers. At least initially, this will challenge algorithm developers to understand clinical information and clinicians to efficiently integrate algorithms into their workflow.

## Abbreviations

AI: artificial intelligence
DARPA: US Defense Advanced Research Projects Agency
XAI: explainable artificial intelligence

## Data Availability

All data generated or analyzed during this study are included in this published article.
